# Cutting the defense budget: How allocation costs shape induced resistance in plants

**DOI:** 10.1371/journal.pbio.3003317

**Published:** 2025-08-15

**Authors:** Ethan Bass

**Affiliations:** Department of Ecology & Evolution, University of Chicago, Chicago, Illinois, United States of America

## Abstract

Plants have limited resources for defense. This Primer explores a new study in PLOS Biology that reveals how plants limit costs through a tiered defense system, deploying cheap traits immediately, while delaying spending on costly ones until a critical damage threshold is reached.

## Teaser

Plants have limited resources for defense. A new study in *PLOS Biology* reveals how plants limit costs through a tiered defense system, deploying cheap traits immediately, while delaying spending on costly ones until a critical damage threshold is reached.

Plants have evolved a variety of strategies to maximize fitness in the face of changing environmental conditions by matching their phenotypes to the environment. For example, plants may produce toxins in response to damage by an herbivore, a classic case of phenotypic plasticity. These induced defenses are thought to evolve as a cost-saving mechanism to prevent the plant from expending resources on defense when herbivores are not present. Theory suggests that reaction norms of induced defenses—how patterns of trait expression change across levels of herbivory—are shaped by the balance of costs and benefits associated with a given trait [1,2]. However, empirical evidence of these relationships is limited due to the difficulty of quantifying or even conceptualizing these costs and benefits, which are highly context-dependent and may involve complex genetic correlations or trade-offs [3,4]. A new *PLOS Biology* study by Wan and colleagues provides new insight into how plants balance resource allocation with effective protection against herbivory through a tiered defense strategy, deploying cheaper traits first and delaying costlier ones until severe damage is detected [5]. Their results suggest that allocation costs are a major factor driving selection on reaction norms of induced defense traits.

**Fig 1:**
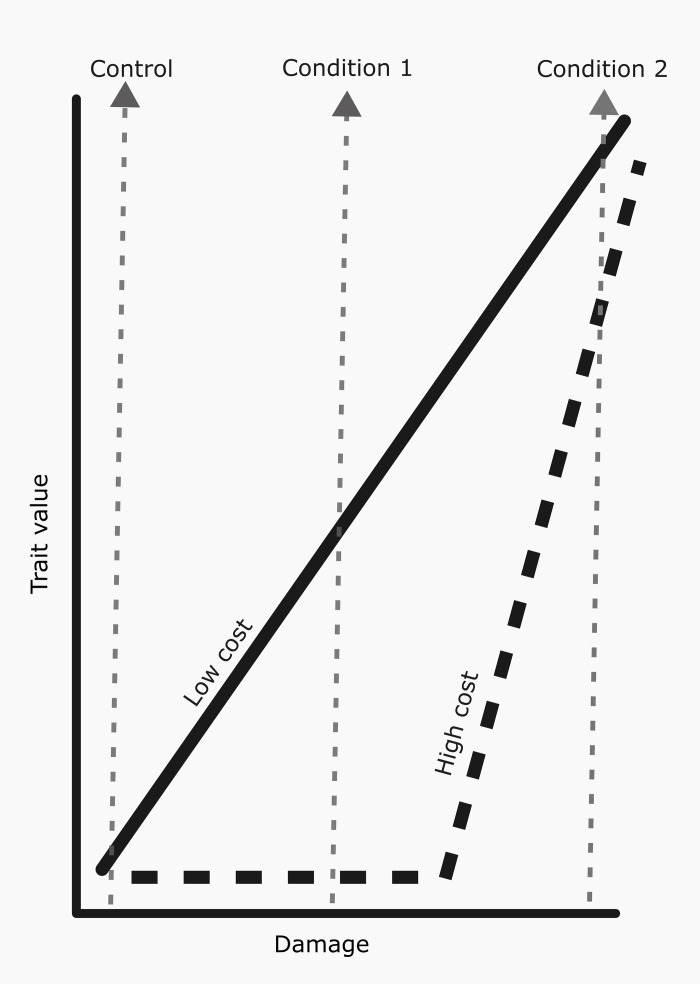
Predicted reaction norms of costly and cheap traits. Schematic of reaction norms for two hypothetical traits: a metabolically cheap trait (e.g., a chemical toxin) with a continuous reaction norm (solid line) and a more expensive trait (e.g., trichome density) with a segmented reaction norm (dotted line). A hypothetical experiment with only two treatments (Control and Condition 1) might erroneously conclude that the segmented trait is fixed. Conversely, an experiment with stronger induction (Condition 2) would find no difference in inducibility between the two traits. By measuring trait values over a gradient of damage, the shapes of the reaction norms can be clearly distinguished. The organization of traits into continuous and threshold defenses may form a tiered defense system that balances cost savings with flexibility against variable threats.

To investigate how allocation costs shape the evolution of induced defenses, Wan and colleagues conducted a series of experiments with the annual plant, common ragweed (*Ambrosia artemisiifolia*) [5]. They hypothesized that traits with higher production costs would have a higher threshold for induction. The authors derive two key predictions from this hypothesis. First, they predicted that structural defenses (e.g., trichomes, which act as a physical deterrent) and large polymers such as condensed tannins and lignin (which typically reduce plant digestibility) are more costly to produce than small molecules such as chlorogenic acid, kaempferol and rutin (which generally function as direct toxins or metabolic inhibitors). Second, they predicted that cheaper traits are induced at lower levels of herbivory compared to more costly ones (Fig 1).

The cost of each trait was first estimated by correlating constitutive expression of each trait with total plant biomass in a common garden where herbivory was excluded. As predicted, evidence of a fitness cost (in the form of reduced growth) was found for trichomes, tannins, and lignin, but not for any of the small molecules. Then, the reaction norms for each trait were estimated by exposing plants from one representative population to a gradient of herbivory by 12 herbivore species. This approach of measuring trait values over a range of environmental conditions is crucial for detecting breakpoints—a simpler study conducted across only two environments would be unable to detect such thresholds and might misclassify traits as fixed or continuously inducible (Fig 1). They found that the induction of the three small molecules was linearly related to damage level, whereas the more costly traits were induced only after a certain damage threshold was exceeded (approximately 40%) [5]. Despite some variation among herbivores, all three of the more costly traits showed evidence of breakpoints, strongly supporting the hypothesis that allocation costs are a key factor shaping the reaction norms of herbivore-induced defenses [5].

The hierarchical defense strategy implied by separation of reaction norms into two distinct lines of defense recalls Paul Feeny’s concept of “quantitative” and “qualitative” defenses [6]. Feeny defined qualitative defenses as small molecules (e.g., glucosinolates) which are effective against many generalist herbivores but can be easily overcome by specialists. In contrast, quantitative defenses (e.g., tannins) are more costly but less susceptible to detoxification [6]. Wan and colleagues’ findings suggest the organization of traits into a tiered defense system, whereby the delayed induction of more costly traits may allow a first line of cheaper (but potentially more easily surmountable) defenses to reduce herbivory before the second line of more costly defenses are deployed. In many cases, this first line of defense may be adequate to repel attacks by non-adapted, generalist herbivores, obviating the need to activate the more costly suite of structural and polymeric defenses.

Overall, the study demonstrates that even a crude approach to measuring production costs (e.g., through phenotypic biomass-trait correlations) can explain patterns of defensive induction, contrary to some theoretical expectations concerning the evolution of plasticity [3]. While its conceptual simplicity is an advantage, this method may overlook other important types of costs, such as opportunity costs and ecological costs, which are often masked in the sheltered environment of a common garden [7]. Trait correlations introduce further complexity. Constitutive expression of trichomes, tannin, and lignin were substantially correlated in the studied populations [5], suggesting that these traits may share a common genetic basis. Exploring these patterns of trait correlations in a multivariate framework may lead to new insights into the evolution of plasticity in integrated defensive phenotypes [8,9].

Another important area for future investigation is the role of specialist herbivores. Wan and colleagues focused only on generalist herbivores, excluding the specialist leaf beetles (*Ophraella communa*) which were identified in a field survey as the dominant herbivores in the study populations [5]. This decision was rationalized because the toxicity of some chemical defenses, such as chlorogenic acid, may differ between generalists and specialists [10], complicating the inference of defensive value [5]. However, dominant herbivores likely exert the strongest selection on defensive traits. Thus, such complications are likely to be an important factor in the evolution of induced defenses. Incorporating specialists such as *O. communa* into future studies would help clarify the relative importance of trait efficacy and cost in shaping the regulation of induced defenses. Future research could extend this work to multiple populations of common ragweed to assess the generality of these findings and how variation in costs and benefits of defensive traits influences the shape of reaction norms across populations.

Despite some limitations, the study provides rare empirical support for a long-standing prediction: that production costs shape not just whether a trait is induced, but when [5]. The results also highlight the importance of moving beyond simple binary induction experiments to understand the precise contours of adaptive responses under variable conditions. By demonstrating how costs shape these reaction norms, Wan and colleagues open new avenues for exploring the broader organization and integration of multivariate plasticity.
